# Pixelwise Phase Unwrapping Based on Ordered Periods Phase Shift

**DOI:** 10.3390/s19020377

**Published:** 2019-01-17

**Authors:** Satoshi Tabata, Michika Maruyama, Yoshihiro Watanabe, Masatoshi Ishikawa

**Affiliations:** 1 Department of Information Physics and Computing, The University of Tokyo, 7-3-1 Hongo, Bunkyo-ku, Tokyo 113-8656, Japan; Michika_Maruyama@ipc.i.u-tokyo.ac.jp; 2 Department of Information and Communications Engineering, School of Engineering, Tokyo Institute of Technology, 4259-G2-31, Nagatsuta, Midori-ku, Yokohama, Kanagawa 226-8502, Japan; watanabe.y.cl@m.titech.ac.jp; 3 Department of Creative Informatics, The University of Tokyo, 7-3-1 Hongo, Bunkyo-ku, Tokyo 113-8656, Japan; Masatoshi_Ishikawa@ipc.i.u-tokyo.ac.jp

**Keywords:** 3D shape measurement, structured light illumination, phase unwrapping, phase shift

## Abstract

The existing phase-shift methods are effective in achieving high-speed, high-precision, high-resolution, real-time shape measurement of moving objects; however, a phase-unwrapping method that can handle the motion of target objects in a real environment and is robust against global illumination as well is yet to be established. Accordingly, a robust and highly accurate method for determining the absolute phase, using a minimum of three steps, is proposed in this study. In this proposed method, an order structure that rearranges the projection pattern for each period of the sine wave is introduced, so that solving the phase unwrapping problem comes down to calculating the pattern order. Using simulation experiments, it has been confirmed that the proposed method can be used in high-speed, high-precision, high-resolution, three-dimensional shape measurements even in situations with high-speed moving objects and presence of global illumination. In this study, an experimental measurement system was configured with a high-speed camera and projector, and real-time measurements were performed with a processing time of 1.05 ms and a throughput of 500 fps.

## 1. Introduction

Three-dimensional (3D) measurement is used in various fields. In particular, achieving high-speed, high-precision, high-resolution real-time shape measurement of moving objects is important in fields requiring high-speed feedback, such as robotics, user interface, virtual reality, and augmented reality. It has been observed that a high-speed measurement, about 500–1000 fps, is effective for accurately measuring a moving object [1]. It has also been observed that a structured light method is effective for ensuring a sufficient amount of light is available in high-speed measurement with short exposure time and for measuring a wide range at once [1]. There are various structured light methods [2,3]. These methods can be classified into two types, one-shot and multi-shot types. The former is a method of projecting one pattern, and the latter is a method of projecting many patterns. Between these two types, the one-shot type with a small number of projection patterns is suitable for measuring moving objects. Although this method increases the computational complexity of pixel correspondence between the projector and the camera, there are some techniques that achieve efficient calculation by encoding the information into the spatial structure of patterns and achieve speed exceeding 500 fps [4,5]. However, it is necessary to structure span a plurality of pixels for one measurement point; thus, the resolution tends to be low. On the other hand, in the multi-shot type, the projection of the plurality of sheets is necessary; thus, the total projection time becomes longer than the one-shot type. However, it can measure independently for each pixel; thus, high resolution can be achieved. Therefore, methods that speed up the Gray code method [6] or the phase-shift method [7] have also been proposed. In particular, the phase-shift method requires a few patterns, and pixel correspondence can be achieved with subpixel accuracy; therefore, the distance can be measured with high accuracy. In addition, an 8-bit projector at 1000 fps has been realized in recent years [8]. The projection time can be reduced, and environments capable of measuring moving objects even in the multi-shot type are available [7].

In the phase-shift method, projecting a minimum of three sine wave patterns, the phase for each pixel of the image captured by the camera is calculated [9]. However, only the wrapped phase can be obtained because the inverse tangent function is used. Accordingly, phase unwrapping is required to obtain the absolute phase. Many phase-unwrapping methods corresponding to various conditions have been proposed [3,10].

There are two important problems in the measurement of moving objects using the phase-shift method. The first one is the motion error. This occurs because of the use of multi-shots. The error is caused by the shift in the projected position as the object moves under measurement. The second one is global illumination. This problem arises as the projected light is reflected in the object under measurement. The intensity of the reflection target changes depending on the intensity of the reflection source when secondary reflection occurs; thus, different intensity changes occur for each projection pattern. These cause error in the phase calculations. This error becomes large when low frequency patterns are used [11].

Methods have been proposed to solve the motion error problem, by motion compensation [12,13], or reducing the number of projections [7,14,15]. Particularly, the approach to reduce the number of projections is considered to be promising to achieve high accuracy with the small calculation amount. For example, two-plus-two method [15] reduced the number of projections and achieved the measurement with only four projections, without any other additional devices. However, this method is easily affected by the global illumination, not only in the calculation of relative phase, but also in the phase unwrapping. On the other hand, to solve the global illumination problem, a method of suppressing the effect by using only high-frequency patterns has been proposed [11]. However, a large number of projection patterns are used in this method, making it unsuitable for measurements of moving objects. Although methods to solve each problem separately have been proposed, there is no known method that solves both problems and can be used in high-speed measurements of moving objects.

Moreover, reducing the processing time is also important to achieve real-time measurement. There is the system which achieved 500 fps based on combination of phase-shift method and high-speed projector [7]. However, this system needs additional camera which placed coaxially, and the camera axes are aligned high-precision.

Accordingly, this study proposes a method to solve these two problems simultaneously without any other additional devices by applying phase unwrapping using an ordered structure of patterns. In this method, unlike the widely used conventional approach of repeating the sine wave patterns, phase unwrapping is achieved by switching the order of the function in each period (2π). This approach is able to reduces the number of projection patterns. That is, with *N* types of functions, the maximum number of switches possible is ${}_{N}P_{N}$, and the period that can be expressed depends on the number of steps. This paper proposes two type of the projection patterns. The first one is for the case with the minimum of three steps, and the second one is for the case with four steps where enough periods can be obtained. Moreover, the proposed method incorporates a robust design to handle the effect of global illumination without using low-frequency patterns.

In the experiments, accuracy comparisons were performed using simulations. The results confirmed that, compared with the conventional methods [15], the proposed method can achieve robust measurements even with global illumination. Moreover, a high-speed system achieving 500 fps (throughput) was realized using a high-speed 8-bit projector [8].

## 2. Related Methods

### 2.1. Basic Method

**Figure 1:**
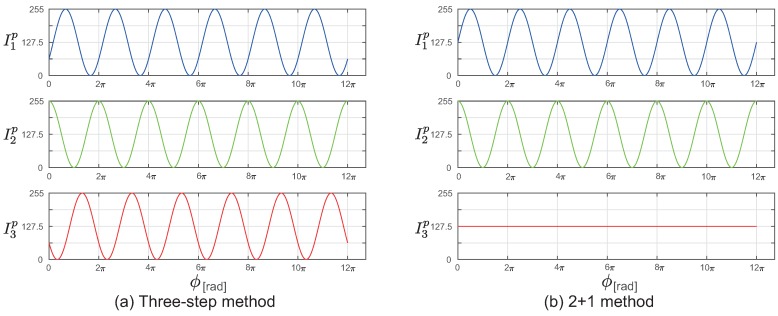
Examples of the projection pattern using the phase-shift method. (**a**) Three-step phase shift and (**b**) Two-plus-one phase shift.

In the phase-shift method, using the number of sine wave patterns *N* (N≥3) projected, the phase of each pixel in the image captured by the camera is calculated, and the shape is measured using the triangulation method. In particular, for N=3, the measurements can be performed with minimum number of steps. In the following, although the method is explained using N=3, the phase can also be calculated for N>3. Figure 1a shows an example of the projection pattern for N=3. This method is called the three-step method. For a projector pixel (u,v), assuming the phase as $\phi^{p}\left(u,v\right)$, the intensity given by $I_{1}^{p}\left(u,v\right)$, $I_{2}^{p}\left(u,v\right)$, and $I_{3}^{p}\left(u,v\right)$ can be expressed as follows:

(1) $$\begin{array}{ccc} I_{1}^{p}\left(u,v\right) & = & I_{DC}^{p}+I_{\phi }^{p}\cos\left(\phi^{p}\left(u,v\right)-\frac{2}{3}\pi \right) \end{array}$$

(2) $$\;\begin{array}{ccc} I_{2}^{p}\left(u,v\right) & = & I_{DC}^{p}+I_{\phi }^{p}\cos\phi^{p}\left(u,v\right) \end{array}$$

(3) $$\begin{array}{ccc} I_{3}^{p}\left(u,v\right) & = & I_{DC}^{p}+I_{\phi }^{p}\cos\left(\phi^{p}\left(u,v\right)+\frac{2}{3}\pi \right) \end{array}$$

where $I_{DC}^{p}$ is the offset value of DC (Direct Current) component and $I_{\phi }^{p}$ is the amplitude of the sine wave.

In the camera pixel (x,y), $I_{1}^{c}$, $I_{2}^{c}$, and $I_{3}^{c}$ are captured after being affected by distance attenuation, object surface reflectance ratio, and ambient light.

(4) $$\begin{array}{ccc} I_{1}^{c}\left(x,y\right) & = & A\left(x,y\right)I_{1}^{p}\left(u,v\right)+B\left(x,y\right) \end{array}$$

(5) $$\begin{array}{ccc} I_{2}^{c}\left(x,y\right) & = & A\left(x,y\right)I_{2}^{p}\left(u,v\right)+B\left(x,y\right) \end{array}$$

(6) $$\begin{array}{ccc} I_{3}^{c}\left(x,y\right) & = & A\left(x,y\right)I_{3}^{p}\left(u,v\right)+B\left(x,y\right) \end{array}$$

where *A* is the attenuation because of the distance and object surface reflectance ratio, and *B* is the ambient light. The distance is determined by calculating the phase ϕ(x,y) at each camera pixel from the captured $I_{1}^{c}$, $I_{2}^{c}$, and $I_{3}^{c}$, and the triangulation measurements corresponding to the projector coordinates using $\phi^{p}$. Although Equation (7) is used in calculating the phase, only the relative phase $\phi^{\prime }\left(0\leq \phi^{\prime }<2\pi \right)$ can be obtained.

(7) $$\phi^{\prime }\left(x,y\right)=\arctan\left(\frac{\sqrt{3}\left(I_{1}^{c}-I_{3}^{c}\right)}{2I_{2}^{c}-I_{1}^{c}-I_{3}^{c}}\right)$$

Accordingly, the absolute phase ϕ needs to be calculated by performing phase unwrapping to calculate the period k∈Z in Equation (8).

(8) $$\phi \left(x,y\right)=\phi^{\prime }\left(x,y\right)+2\pi k\left(x,y\right)$$

Various methods have been proposed not only using the sine wave as the projection pattern but also using the triangular [16] and trapezoidal waves [17]. However, these patterns are prone to defocused blur [18,19], and it is observed that using sine wave patterns is preferable.

Moreover, to suppress the motion error, the two-plus-one phase-shift method, in which one step is taken as steady light, has been proposed [14]. In this method, instead of Equations (1)–(3) of the three-step method,

(9) $$\begin{array}{ccc} I_{1}^{p}\left(u,v\right) & = & I_{DC}^{p}+I_{\phi }^{p}\sin\phi^{p}\left(u,v\right) \end{array}$$

(10) $$\begin{array}{ccc} I_{2}^{p}\left(u,v\right) & = & I_{DC}^{p}+I_{\phi }^{p}\cos\phi^{p}\left(u,v\right) \end{array}$$

(11) $$\;\begin{array}{ccc} I_{3}^{p}\left(u,v\right) & = & I_{DC}^{p} \end{array}$$

are projected. Figure 1b shows the projection pattern in the two-plus-one phase-shift method. The relative phase of each pixel in the camera is calculated using the following equation with these values.

(12) $$\phi^{\prime }\left(x,y\right)=\arctan\left(\frac{I_{1}^{c}-I_{3}^{c}}{I_{2}^{c}-I_{3}^{c}}\right)$$

In this method, Equation (11) forms a uniform pattern for the entire projector; thus, it is not affected by the projected position change caused by motion. This way the effect of the motion error can be suppressed. However, similarly to the three-step method, this method also requires phase unwrapping, as presented in Equation (8).

The relative phase and absolute phase coincide as the only projected phase is 0≤ϕ<2π, and this phase can be identified without performing phase unwrapping. However, the spatial resolution is severely limited because of the limitations of intensity, such as gradation. Therefore, phase unwrapping becomes necessary. The following describes this in detail.

### 2.2. Phase Unwrapping

In phase unwrapping, the two broad approaches are the spatial and temporal methods [10]. This section summarizes the characteristics, merits, and demerits of these methods. First, in the spatial phase-unwrapping method, phase unwrapping is performed utilizing the spatial phase continuity without increasing the number of projections, and accordingly, various methods have been proposed [20,21,22,23]. Although the spatial phase-unwrapping method can measure a single continuous shape, it is prone to failure in case of multiple objects or discontinuous changes. Thus, the measurement targets are limited. Furthermore, there are single-shot type of spatial methods for the fringe pattern [24,25]. Although these are not proposed for the phase-shift method, it could be applied to the phase-shift method. These methods are able to be applied in case of multiple objects, because each pixel only depends on small patch. However, there are some error and blur around the discontinuous changes, since they are not pixelwise methods.

In contrast, in the temporal phase-unwrapping method, pixelwise phase unwrapping can be performed using additional projections in addition to the three steps, which is the minimum number of projections required to calculate the relative phase [26]. In the temporal phase-unwrapping method, there is no limitation to the additional projection patterns, and various patterns, such as multi-frequency and Gray-code, have been proposed [11,15,27,28,29,30,31]. It is important to note that, depending on the projection pattern type, the number of additional projections varies. For example, in the multi-frequency method, 3F additional projections are required if the number of frequencies to be added is *F*; and for the Gray-code pattern, *F* projections are required to express $2^{F}$ periods. However, if the number of projections increases, it takes longer to project all the patterns resulting in slower measurement speed. In particular, in measuring moving objects, the movement distance during projection becomes longer, resulting in increase in error caused by motion. To solve such problems associated with an increase in the number of projections in phase unwrapping, a measurement method using four steps has been proposed [15]. This method is based on the two-plus-one phase-shift method [14], utilizing projections using Equations (9)–(11). Thus, instead of Equation (11), this method uses projection patterns increasing or decreasing monotonically and identifies the period based on the magnitude of the difference. However, the monotonically increasing or decreasing projection patterns can be affected by global illumination and period misidentification. Although methods have been proposed to reduce the number of projections by distributing each pattern in RGB and projecting them simultaneously [31,32,33,34], in all these methods, the measurement accuracy depends on the surface color of the target object. Moreover, a method has also been proposed in which the phase-shift pattern is expressed in seven bits and the Gray-code pattern is expressed in one bit [7]; and by adjusting the projection time for each bit of the projector, the patterns are embedded in one image [7]. However, two cameras should be placed coaxially to capture the respective patterns, which makes the system complex and calls for high-precision alignment of the camera axes.

Other than the aforementioned methods, various phase-unwrapping methods incorporating geometrical constraints using a number of cameras [35,36], or limiting the range of measurement [37], have also been proposed. However, the methods based on multiple cameras need long processing time, bacause they need global search to select the correct corresponding point [37], and the methods based on limiting the range of measurement can measure only a narrow area.

Moreover, a method has been proposed in which the amplitude of conventional phase-shift method pattern changes for each period, and phase unwrapping is performed by identifying the phase from the magnitude of the amplitude [38]. Although the period can be identified using only the projected pattern without increasing the number of projections required for phase unwrapping, pixelwise phase unwrapping has not been achieved because using a spatial codeword is necessary because of the limitations of amplitude resolution.

As opposed to the aforementioned spatial methods, the proposed method can be applied in case of multiple objects or discontinuous changes without blur of depth, because pixelwise phase unwrapping can be performed in the proposed method. Moreover, as opposed to the aforementioned temporal methods, the proposed method can also be applied to moving objects, because phase unwrapping can be performed using as few as three or four steps in the proposed method. Furthermore, by making the period identification condition used in phase unwrapping robust enough against intensity changes, stable application in cases with global illumination is also possible. Also, because no extra cameras or limiting the measurement range is necessary, the method enables application to a wide range of measurements using a simple system.

## 3. Proposed Method

Many conventional methods use a function that repeats the same value for each period in each image of the projection pattern. Therefore, the projection pattern satisfies Equation (13).

(13) $$I_{n}^{p}\left(\phi \right)=I_{n}^{p}\left(\phi +2\pi k\right)\;\left\{\forall n|n=1\cdots N\right\},k\in Z$$

In contrast to conventional methods, the proposed method rearranges part of the projection pattern for each period among the images of the projection patterns. In this way, as described in the Section 3.1.1, each function is arranged in a different order. This structure is called “ordered periods” in this paper.

With this structure, phase unwrapping is solved from what order each part of each projection pattern is projected. In next subsection, the procedure of designing the proposed method and the actual projection pattern for three-step and four-step methods are described.

### 3.1. Ordered Periods Phase Shift (OPPS)

#### 3.1.1. Basic Method

First, this subsection introduces about the procedure of designing the OPPS method. Let $I_{n}^{\prime }\left(n=1\cdots N\right)$ be one period of the cyclic *N* patterns, which used in the standard methods such as three-step method or two-plus-one method. With dividing *M* periods into 1st period ([0,2π)), 2nd period ([2π,4π)),…, and *M*th period ([(M−1)·2π,M·2π)), *N* patterns of OPPS are denoted as

(14) $$I_{n}^{p}\left(\phi^{p}\right)=\left\{\begin{array}{cc} I_{S_{n}^{1}}^{\prime }\left(\phi^{p}\right) & \left(0\leq \phi^{p}<2\pi \right)\\ \vdots \\ I_{S_{n}^{m}}^{\prime }\left(\phi^{p}\right) & \left(\left(m-1\right)\cdot 2\pi \leq \phi^{p}<m\cdot 2\pi \right)\\ \vdots \\ I_{S_{n}^{M}}^{\prime }\left(\phi^{p}\right) & \left(\left(M-1\right)\cdot 2\pi \leq \phi^{p}<M\cdot 2\pi \right) \end{array}\right.$$

where *S* is rearrangement of s={1,⋯,N}, $S^{m}$ is *m*th tuple of *S*, and $S_{n}^{m}$ is *n*th element of $S^{m}$.

In the case of N=3, let the rearrangement be

(15) S={(1,2,3),(1,3,2),(2,1,3),(2,3,1),(3,1,2),(3,2,1)}

Further, these elements are $S_{1}^{1}=1$, $S_{1}^{2}=1$, and $S_{2}^{1}=2$; and $I_{S_{1}^{1}}^{\prime }=I_{1}^{\prime }$, $I_{S_{1}^{2}}^{\prime }=I_{1}^{\prime }$, and $I_{S_{2}^{1}}^{\prime }=I_{2}^{\prime }$. With the patterns designed in such procedure, phase unwrapping problem results into the problem of identifying the tuple in each pixel.

Here, the three-step OPPS pattern is introduced. For example, for the three-step OPPS pattern, Equations (1)–(3) for three-step method, or Equations (9)–(11) for two-plus-one method, can be considered as standard pattern. However, $I_{1}^{\prime }$, $I_{2}^{\prime }$, and $I_{3}^{\prime }$ of three-step method have same intensity pair with phase shift of $\frac{2}{3}\pi$, and $I_{1}^{\prime }$ and $I_{2}^{\prime }$ of two-plus-one method have it with phase shift of $\frac{\pi }{2}$. Therefore, with the rearrangement *S* (Equation (15)), large areas have same intensity pair in different tuples. Accordingly, it is necessary to omit the areas with duplicate intensity to appropriately determine the absolute phase, but it becomes unsuitable as the usable range of absolute phase becomes smaller. Therefore, in this study, the following are used.

(16) $$\begin{array}{ccc} I_{1}^{\prime }\left(u,v\right) & = & I_{DC}^{\prime }+I_{\phi }^{\prime }\cos\phi^{\prime }\left(u,v\right) \end{array}$$

(17) $$\;\begin{array}{ccc} I_{2}^{\prime }\left(u,v\right) & = & 2I_{DC}^{\prime } \end{array}$$

(18) $$\;\begin{array}{ccc} I_{3}^{\prime }\left(u,v\right) & = & 0 \end{array}$$

Here, $I_{DC}^{\prime }$ is the offset value of DC (Direct Current) component and $I_{\phi }^{\prime }$ is the amplitude of the sine wave. If $I_{DC}^{\prime }$ and $I_{\phi }^{\prime }$ satisfy $0<I_{\phi }^{\prime }<I_{DC}^{\prime }$, this pattern satisfies $I_{3}^{\prime }<I_{1}^{\prime }<I_{2}^{\prime }$ everywhere.

An ordered structure is incorporated in this pattern. In case of three-step, because the maximum number of tuples shown in Equation (15) exists, expressing up to six periods is possible. Therefore, creating $I_{n}^{p}\left(\phi^{p}\right)$ as shown in Equation (14), it can be used up to 12π. However, there are certain problems in using this in actual measurements. Accordingly, patterns obtained by rearranging Equation (14) are used. The rearrangement of Equation (14) is described in the following subsections.

#### 3.1.2. Realizing Unique Tuple Identification

First, as represented by Equations (4)–(6), the camera pixel intensity is actually measured only after being affected by distance attenuation, object surface reflectance ratio, and ambient light. Accordingly, it is necessary to uniquely identify the tuple only from this value, using the relative magnitude or comparisons. The essential condition for uniquely identifying one tuple is that any set of $\left\{I_{1}^{c},I_{2}^{c},I_{3}^{c},...I_{N}^{c}\right\}$ in it does not appear in any other tuples. Accordingly, among the $I_{n}^{p}\left(\phi^{p}\right)$ created using rearrangement *S*, for the areas satisfying Equation (19), patterns are omitted to enable unique tuple identification.

(19) $$\overset{N}{\underset{n=1}{⋀}}\left|I_{n}^{c}\left(\phi \right)-I_{n}^{c}\left(\theta \right)\right|<\epsilon \;\;\;\;\left\{0<\theta <2M\pi \right\}$$

where ϵ is the difference in value to consider the intensity to be identical, which is set on the basis of the intensity of noise acquired in the real system. This way, by updating the *N* number of projection patterns $I_{n}^{p}\left(n=1\cdots N\right)$, phase unwrapping can be performed by calculating the period because the tuple can be identified only from the pixelwise intensity.

**Figure 2:**
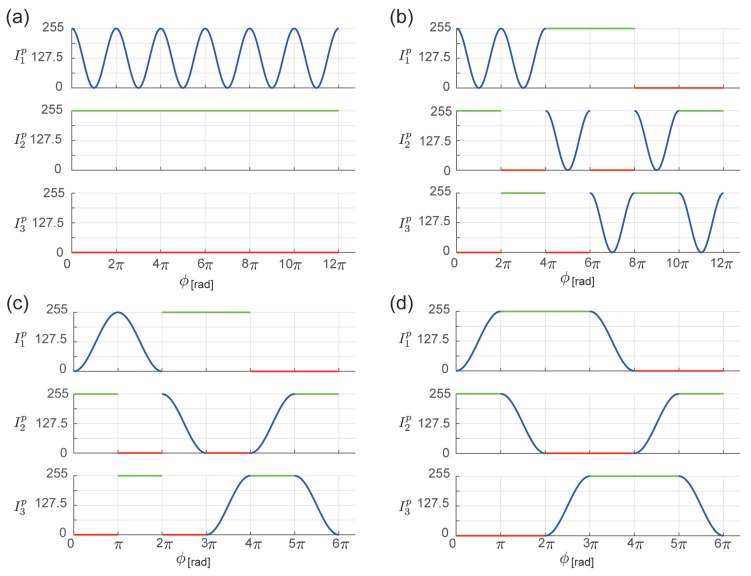
Design of projection pattern for the three-step OPPS. (**a**) Standard projection patterns $I_{1}^{p}$, $I_{2}^{p}$, and $I_{3}^{p}$. (**b**) Incorporating ordered structure (Section 3.1.1). Three steps are reordered; thus, there are ${}_{3}P_{3}=6$ combinations. (**c**) Constrained to uniquely identify tuple (Section 3.1.2). (**d**) Spatial continuity (Section 3.1.3).

The following holds for Equations (16)–(18) used in this study; thus,

(20) cosϕ=cos(2π−ϕ)

for each period, only one range from $0\leq \phi^{\prime }<\pi$ and $\pi \leq \phi^{\prime }<2\pi$ can be used. This selection is used in each period considering spatial continuity, as described in the following subsection. The projection patterns designed in this manner are shown in Figure 2c.

#### 3.1.3. Spatial Continuity of Patterns

Let us consider the problem in which an intermediate intensity value is generated because of light entering from more than two projector pixels into one pixel in the camera. Different patterns are projected for each period in the proposed method; thus, the part $\phi^{p}=2k\pi \left(k\in Z\right)$, where the period is unwrapped (connected), can be discontinuous. Around this discontinuous part, the intermediate intensity value deviates largely from the original intensity. That may lead to wrong results.

Accordingly, the projection patterns are improved to be continuous everywhere, to eliminate this error. In the proposed phase-unwrapping method, the processing for the projection patterns are independently done at each pixel; thus, even if the rearrangement is done considering the internal coordinate system (u,v) of the projection patterns, the tuple can be identified without any problem. Accordingly, the projection pattern $I_{n}^{p}\left(\phi^{p}\right)$ is divided into parts at constant intervals, and spatial rearrangement is performed so that the intensity change is continuous at the period unwrapping part. The rearrangement information is stored in a table, and the phase is transformed by referring to this table at the time of measurement. There are many candidate procedures for this rearrangement. Although spatial continuity can be achieved using multiple rearrangement methods, it is desirable that the average partial intensity be constant over *N* projections, so that the effect of global illumination can be suppressed.

In Equations (16)–(18) used in this study, spatial continuity is achieved by dividing the entire range at intervals of π and rearranging them. First, index κ(κ=1,...,6) is assigned so that Equation (21) is satisfied at the divided regions.

(21) $$\left(\kappa -1\right)\pi \leq \phi^{p}<\kappa \pi$$

Further, the regions assigned with index κ are rearranged using the data shown in Table 1. Using this processing, the patterns shown in Figure 2d are obtained.

Finally, the entire range of $0\leq \phi^{p}<6\pi$ can be projected. Moreover, it is possible to suppress the motion error because the total number of projection steps is three, and $I_{1}^{\prime }\left(u,v\right)$ is the only term that contains $\phi^{\prime }$ which may cause a phase error because of the effects of motion.

#### 3.1.4. Decoding Projected Patterns

In this subsection, the decoding method is described. This method is applied in measurements using the resultant patterns. Even when $I_{1}^{c}$, $I_{2}^{c}$, and $I_{3}^{c}$ at the camera are obtained using Equations (4)–(6), the relative magnitude order $I_{3}^{\prime }<I_{1}^{\prime }<I_{2}^{\prime }$ as determined later using Equations (16)–(18) does not change. Therefore, the relative phase is given by,

(22) $$\phi^{\prime }=\arccos\left(\left(2\cdot \frac{I_{\mathrm{mid}}^{c}-I_{\min}^{c}}{I_{\max}^{c}-I_{\min}^{c}}-1\right)\frac{I_{DC}^{\prime }}{I_{\phi }^{\prime }}\right)$$

where $I_{\mathrm{mid}}^{c}$, $I_{\max}^{c}$, and $I_{\min}^{c}$ are the middle, maximum, and minimum values of $\left\{I_{1}^{c},I_{2}^{c},I_{3}^{c}\right\}$, respectively. Moreover, before rearranging the intensity by magnitude, index κ in Figure 2c can be identified, as shown in Table 2.

Referring to Table 1 with the index κ obtained from Table 2, the absolute phase can be calculated as

(23) $$\phi =\left\{\begin{array}{cc} \phi^{\prime }+\left(\mathrm{hash}\left(\kappa \right)-1\right)\cdot \pi & \left(\kappa =2,3,6\right)\\ \left(\pi -\phi^{\prime }\right)+\left(\mathrm{hash}\left(\kappa \right)-1\right)\cdot \pi & \left(\kappa =1,4,5\right) \end{array}\right.$$

where grouping is done based on κ because 0≤arccosθ<π.

In the case of the measurement of moving objects, the projected position of each pixel change among $I_{1}^{c},I_{2}^{c},$ and $I_{3}^{c}$. Therefore, phase unwrapping may fail around the connection point of two regions which assigned with different indexes κ. However, phase unwrapping don’t fail inside the each region, because the relative magnitude order of $\left\{I_{1}^{c},I_{2}^{c},I_{3}^{c}\right\}$ is same inside the each region.

#### 3.1.5. Measurement Interpolation

In the three-step OPPS, only Equation (16) has the phase information; thus, for each pixel, the distance corresponding to the timing of projection of $I_{1}^{\prime }$ is measured. Accordingly, the distance is measured for the timings that are different at different parts of the projection pattern. In case of a high frame rate of measurement, the velocity change before and after the measurement can be considered to be very small. Accordingly, by interpolating the distance from before and after measurements, measurements can be performed without lowering the frame rate.

#### 3.1.6. Projection Period Limitations

Based on the aforementioned design, no additional projection is required for phase unwrapping in OPPS; thus, measurements can be performed with the minimum of three steps. Fewer steps are preferable to suppress the effect of motion error. However, the periods over which the ordered structure of three steps can be defined are fewer; thus, in cases where more periods are required, use of four steps is preferable. Accordingly, the design of the four-step OPPS is described in the following section.

### 3.2. Four-Step OPPS

#### 3.2.1. Basic Method

**Figure 3:**
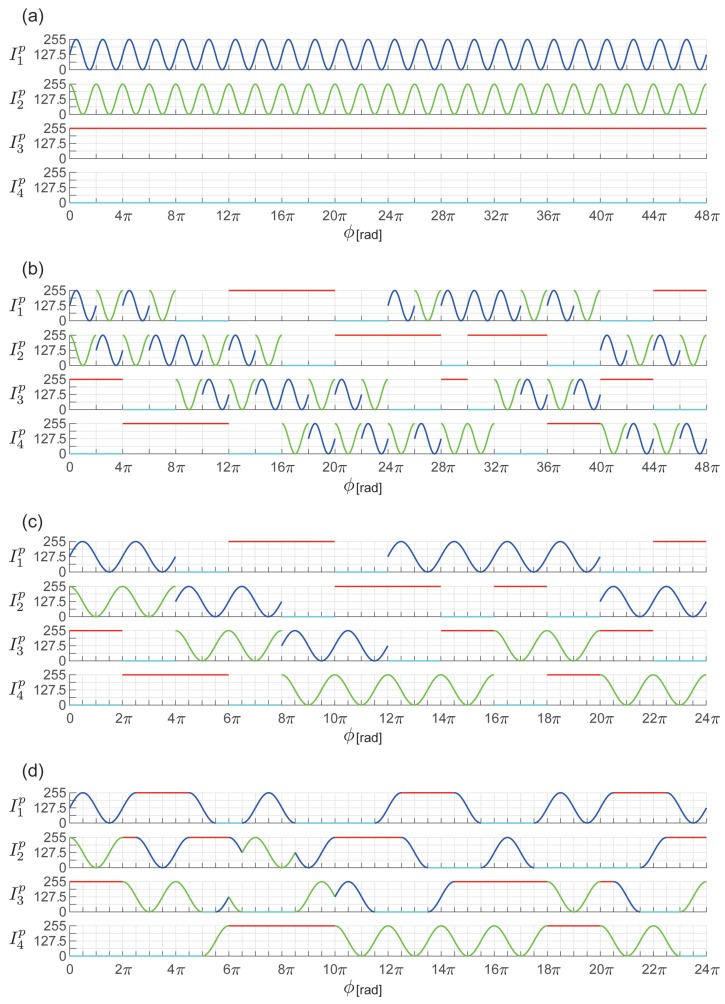
Design of projection pattern for the four-step OPPS. (**a**) Standard projection patterns $I_{1}^{p}$, $I_{2}^{p}$, $I_{3}^{p}$, and $I_{4}^{p}$. (**b**) Incorporating ordered structure (Section 3.2.1). Four steps are reordered; thus, there are ${}_{4}P_{4}=24$ combinations. (**c**) Constrained to uniquely identifying tuple (Section 3.2.2). (**d**) Spatial continuity (Section 3.2.3).

First, as the standard pattern for the four-step OPPS, the following extensions of Equations (9)–(11) of two-plus-one method are used (Figure 3a).

(24) $$\begin{array}{ccc} I_{1}^{\prime }\left(u,v\right) & = & I_{DC}^{\prime }+I_{\phi }^{\prime }\sin\phi^{\prime }\left(u,v\right) \end{array}$$

(25) $$\begin{array}{ccc} I_{2}^{\prime }\left(u,v\right) & = & I_{DC}^{\prime }+I_{\phi }^{\prime }\cos\phi^{\prime }\left(u,v\right) \end{array}$$

(26) $$\;\begin{array}{ccc} I_{3}^{\prime }\left(u,v\right) & = & 2I_{DC}^{\prime } \end{array}$$

(27) $$\;\begin{array}{ccc} I_{4}^{\prime }\left(u,v\right) & = & 0 \end{array}$$

where $I_{DC}^{\prime }$ is the offset value of DC (Direct Current) component and $I_{\phi }^{\prime }$ is the amplitude of the sine wave. An ordered structure is incorporated into this pattern. For the four steps, as presented in Equation (28), there are maximum ${}_{4}P_{4}=24$ combinations for the rearrangement (Figure 3b).

(28) $$\begin{array}{c} S=\{(1,2,3,4),(2,1,3,4),(1,2,4,3),(2,1,4,3),(4,1,2,3),(4,2,1,3),\\ (3,1,2,4),(3,2,1,4),(3,4,1,2),(3,4,2,1),(4,3,1,2),(4,3,2,1),\\ (1,3,4,2),(2,3,4,1),(1,4,3,2),(1,3,4,2),(1,3,2,4),(2,3,1,4),\\ \left(1,4,2,3),(2,4,1,3),(4,1,3,2),(4,2,3,1),(3,1,4,2),(3,2,4,1)\right\} \end{array}$$

#### 3.2.2. Realizing Unique Tuple Identification

Similarly to the three-step OPPS, the pattern is improved to enable unique tuple identification. In Equations (24)–(27), sinθ and cosθ have a phase difference of $\frac{\pi }{2}$. Therefore, in rearranging Equation (28), the rearrangements of $I_{1}^{\prime }$ and $I_{2}^{\prime }$ are omitted. This enables expression up to 12 periods. Accordingly, by creating $I_{n}^{p}\left(\phi^{p}\right)$ as shown in Equation (14), it can be used up to 24π. The resultant projection patterns are shown in Figure 3c.

#### 3.2.3. Spatial Continuity of Patterns

Further, continuity is enforced because there is discontinuity in the period unwrapping part. Using Equations (24)–(27), spatial continuity is achieved by dividing the whole range into intervals of $\frac{\pi }{2}$ and rearranging them. First, index κ(κ=1,…,48) is assigned so that Equation (29) is satisfied at the divided regions.

(29) $$\left(\kappa -1\right)\cdot \frac{\pi }{2}\leq \phi^{p}<\kappa \cdot \frac{\pi }{2}$$

The regions assigned with index κ are then rearranged using the data shown in Table 3. Using this processing, the patterns shown in Figure 3d are obtained. There are various ways of rearrangement to enforce continuity. In this study, to suppress the effect of global simulation, the rearrangement was selected for which the differences between the images were minimum, after applying a median filter.

Finally, the projection can be done for the entire range of $0\leq \phi^{p}<24\pi$. Moreover, $I_{1}^{\prime }\left(u,v\right)$ and $I_{2}^{\prime }\left(u,v\right)$ contain $\phi^{\prime }$ which may cause phase error because of the effects of motion, and it is to be noted that depending on which order these are projected, the magnitude of motion error varies.

#### 3.2.4. Decoding Projection Patterns

In this subsection, the decoding method for four-step OPPS is described. This method is applied in measurements using the patterns created as described in Section 3.2.1, Section 3.2.2 and Section 3.2.3. With respect to $I_{1}^{\prime }$, $I_{2}^{\prime }$, $I_{3}^{\prime }$, and $I_{4}^{\prime }$,

(30) $$\begin{array}{c} I_{3}^{\prime }=\max\left\{I_{1}^{\prime },I_{2}^{\prime },I_{3}^{\prime },I_{4}^{\prime }\right\} \end{array}$$

(31) $$\begin{array}{c} I_{4}^{\prime }=\min\left\{I_{1}^{\prime },I_{2}^{\prime },I_{3}^{\prime },I_{4}^{\prime }\right\} \end{array}$$

always hold. Accordingly, based on the relative magnitude of $\left\{I_{1}^{c},I_{2}^{c},I_{3}^{c},I_{4}^{c}\right\}$, the tuple can be identified as shown in Table 4. Moreover, the corresponding tuple index $\kappa_{\mathrm{tuple}}$ is determined as given in Table 4. At this time, the relative phase given by,

(32) $$\phi^{\prime }=\arctan\left(\frac{2I_{\sin}^{c}-\left(I_{\max}^{c}+I_{\min}^{c}\right)}{2I_{\cos}^{c}-\left(I_{\max}^{c}+I_{\min}^{c}\right)}\right)$$

where $I_{\max}^{c}$ and $I_{\min}^{c}$ are maximum and minimum values, respectively, and $I_{\sin}^{c}$ and $I_{\cos}^{c}$ are determined as given in Table 4.

Moreover, the region is divided into intervals of π/2, as given by Equation (29), and then rearranged as presented in Table 3. Accordingly, depending on the phase quadrant of $\phi^{\prime }$, the quadrant index $\kappa_{\phi }$ is defined as follows:

(33) $$\kappa_{\phi }=\left\{\begin{array}{cc} 1 & \left(0\leq \phi^{\prime }<\frac{\pi }{2}\right)\\ 2 & \left(\frac{\pi }{2}\leq \phi^{\prime }<\pi \right)\\ 3 & \left(\pi \leq \phi^{\prime }<\frac{3}{2}\pi \right)\\ 4 & \left(\frac{3}{2}\pi \leq \phi^{\prime }<2\pi \right) \end{array}\right.$$

Here, the index κ in Figure 3c, before undertaking the spatial continuity procedure described in Section 3.2.3, is obtained as given below.

(34) $$\kappa =4\kappa_{\mathrm{tuple}}+\kappa_{\phi }$$

Using this index κ, and Table 3, the absolute phase is calculated as follows.

(35) $$\phi =\phi^{\prime }-\frac{\pi }{2}\kappa_{\phi }+\mathrm{hash}\left(\kappa \right)\cdot \frac{\pi }{2}$$

## 4. Experiments

### 4.1. Evaluation of Motion Error

The proposed method enables measurement using few steps, and because the number of steps affected by motion is few, the effects of motion associated with moving targets can be suppressed. To verify this, the measurement error under moving conditions was evaluated using simulation utilizing OpenGL. The comparison targets were the proposed three-step and four-step methods, one period of the two-plus-one method (2 + 1) [14] that does not need phase unwrapping, and the two-plus-two method (2 + 2) [15] with temporal phase unwrapping needing the minimum number of steps. Here, the projection period used in the 2 + 2 method was the same 24π as used in the proposed four-step method. Moreover, in the 2 + 2 method, the error changes because of motion in between the two sine wave projection patterns with non-monotonous changes. Accordingly, as the four patterns are projected cyclically, and when the measurements are performed at the same frame rate as the projector, in each frame, the order of the four patterns differ which results in the difference in magnitude of the error. Therefore, the respective errors were calculated for the case of $\left(I_{1},I_{2},I_{3},I_{4}\right)$, $\left(I_{2},I_{3},I_{4},I_{1}\right)$, $\left(I_{3},I_{4},I_{1},I_{2}\right)$, and $\left(I_{4},I_{1},I_{2},I_{3}\right)$ (abbreviated as 2 + 2(1234), 2 + 2(2341), 2 + 2(3412), and 2 + 2(4123), respectively) and the corresponding average value (2 + 2(ave)).

A planar surface as the measurement target was placed at a distance of d=600[mm] from the camera. With the position of the camera as the origin of the coordinate system, the projector was placed at the position (0, 200, and 50 mm), pointing to the position (0, 0, and 600 mm). Moreover, in the experiments, the resolution of the camera and the projector was set as 1024 × 768, and the respective internal parameters, $K_{\mathrm{cam}}$ and $K_{\mathrm{proj}}$, were set as follows.

(36) $$K_{\mathrm{cam}}=\left(\begin{array}{ccc} 1600 & 0 & 512\\ 0 & 1600 & 384\\ 0 & 0 & 1 \end{array}\right),K_{\mathrm{proj}}=\left(\begin{array}{ccc} 1911 & 0 & 512\\ 0 & 1433 & 384\\ 0 & 0 & 1 \end{array}\right)$$

**Figure 4:**
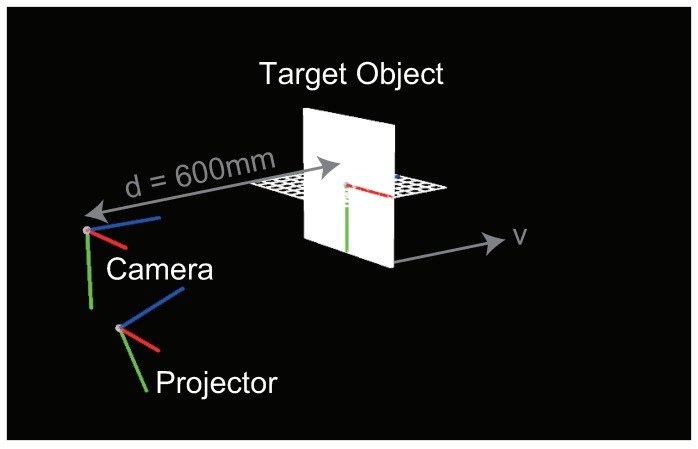
Experimental configuration for simulation.

Figure 4 shows the experimental configuration for simulation, including the placement of the camera and the projector. Here, to separate the effect of global illumination, the rendering was done assuming absence of secondary reflection.

Measurements were conducted with this experimental configuration, by changing the noise magnitude and motion velocity, and under each condition, the distance error was calculated. Here, the noise was applied at three different levels of σ=0%,1%, and 5% corresponding to the intensity. Moreover, the direction of motion was set as v=(0,0,1)[mm/frame], and the measurements were performed corresponding to each noise setting by changing the velocity. Also, the real value was taken as the position of the planar surface at the median time ($\frac{N}{2}$) among the *N* projections used in each of the methods, and the root mean square error (RMSE) calculated from this planar surface was taken as the error. Here, the outliers because of erroneous period were excluded.

**Figure 5:**
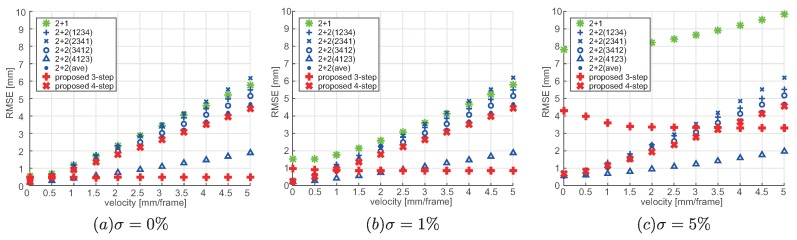
Motion errors. (**a**) RMSE for noise σ=0%, (**b**) RMSE for noise σ=1%, and (**c**) RMSE for noise σ=5%.

Figure 5 shows the motion error for each of the methods under each of the conditions.

First, as shown in Figure 5a, under the condition of no noise, as the velocity increases, the error increases for the methods in the order of proposed method (three-step), 2 + 2(4123), proposed method (four-step), 2 + 2(ave), 2 + 2(3412), 2 + 2(1234), 2 + 1, and 2 + 2(2341).

In particular, in case of the proposed method (three-step) the error is constant and does not change with velocity. This is because the proposed method (three-step) is not affected by motion.

Moreover, in the 2 + 2 method, $I_{1}^{p}$ and $I_{2}^{p}$ mainly include $\phi^{\prime }$ and thereby affected by motion; thus, for the 2 + 2(4123) that falls in between these projections the error is the lowest. There is only one frame in between $I_{1}^{p}$ and $I_{2}^{p}$, and it is relatively robust against motion. In case of 2 + 2(3412) and 2 + 2(1234), there is a symmetrical deviation corresponding to the real value at the planar surface based on the order of $I_{1}^{p}$ and $I_{2}^{p}$, and the level of error is similar. In contrast, in case of 2 + 2(2341), there are three frames in between $I_{1}^{p}$ and $I_{2}^{p}$, and the error is especially large.

Moreover, the proposed method (four-step) and 2 + 2(ave) show similar levels of error. This may be because of the fact that the resolutions are similar, excluding the effects of phase unwrapping and global illumination, because the number of periods projected is the same. Moreover, the error in 2 + 2(4123) is lower than that in the proposed method (four-step). However, in case of cyclic projection, the frame rate is 1/4 of the proposed method (four-step). Moreover, in the proposed method (four-step), for each region, the order of $I_{1}^{p}$ and $I_{2}^{p}$ can be obtained. If the measurement timing is corrected using this information, error level is similar to that of 2 + 2(4123) can be achieved. Moreover, as it can be considered as measurement at different regions at different points in time, it is likely that highly accurate reproduction of shape is possible by incorporating spatial and temporal corrections.

As shown in Figure 5b,c, as the noise increases, in particular, the errors become larger in the proposed method (three-step) and 2 + 1 method. This is because the smaller the number of periods, the larger is the effect on the phase when the intensity varies by 1. Accordingly, in case of noise of σ=5%, the error caused by noise becomes larger than the error caused by motion, showing larger error in the proposed (three-step) method.

Finally, based on the aforementioned results, the way of selecting proposed methods is able to be described; thus, it can be presumed that the proposed method (three-step) with fewer number of steps is suitable in cases where the effect of motion is dominant, whereas the proposed method (four-step) with larger number of periods is suitable in cases where the effect of noise is dominant.

### 4.2. Evaluation of Global Illumination

Next simulation experiments were conducted to measure the effect of global illumination. Here, assuming absence of motion, the error was evaluated by rendering using *cycles render* in *blender 2.79a* [39] to simulate global illumination. Moreover, the comparison targets were the proposed three-step, four-step methods, and the 2 + 2 method.

The camera and the projector were placed coaxially, because mainly the error in phase unwrapping is evaluated in this study. Therefore, the error is calculated from the absolute phases which measured by each methods; thus, the phase difference with the ground truth was taken as the error. Two planar surfaces intersecting at 90 degrees and a Stanford Bunny were placed in front of the camera as measurement targets.

**Figure 6:**
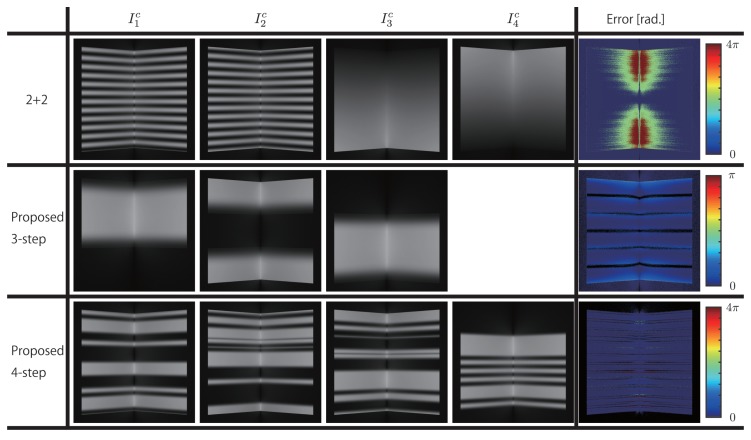
Measurement results for two planar surfaces intersecting at 90 degrees.

**Figure 7:**
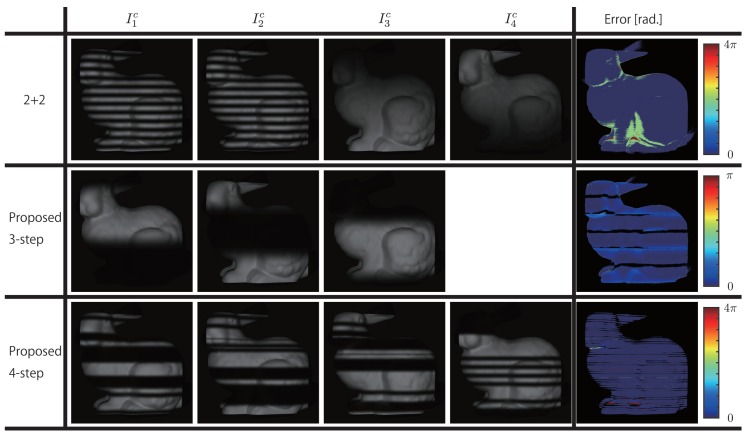
Measurement results for Stanford Bunny.

The measurement results for the planar surfaces intersecting at 90 degrees are shown in Figure 6, and the results for the Stanford Bunny are shown in Figure 7. Here, the magnitude of the error is expressed using blue (0[rad.])−red (4π[rad.]) for 2 + 2 and proposed four-step methods where 24π is used, and using blue (0[rad.])−red (π[rad.]) for the proposed three-step method where 6π is used. Moreover, the areas that are not measured are not considered for error calculation and are shown in black.

As shown in Figure 6, in the 2 + 2 method, period error is generated in the areas above and below the central part of the image. In particular, in the concave part, the errors are larger. In contrast, in both the proposed three-step and four-step methods, excluding the period boundaries, no period error is generated. However, phase errors are generated to some extent, because the effect itself—a result of intensity changes caused by global illumination—cannot be avoided. Moreover, the magnitude of phase error for the proposed three-step method with fewer periods is larger than that for the proposed four-step method. As shown in Figure 7, similar errors in the concave areas of the Stanford Bunny, especially period errors, are generated in the 2 + 2 method.

### 4.3. High-Speed OPPS

**Figure 8:**
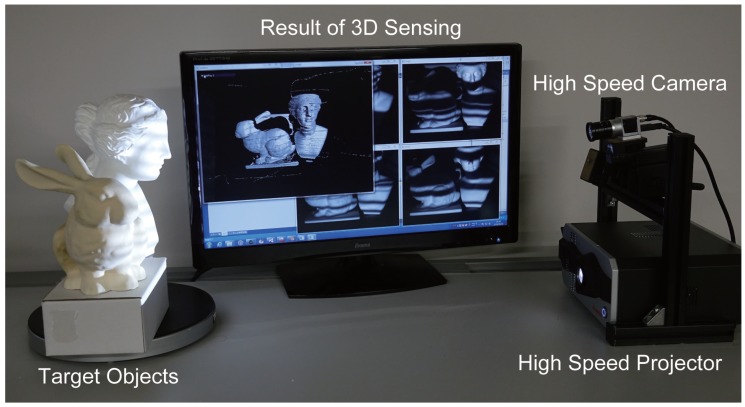
Experimental environment. The high-speed projector and high-speed camera were synchronized, and the camera was placed vertically above the projector to perform the measurements. The measurement results were obtained in real-time and displayed in the monitor screen.

To conduct actual measurements using the proposed methods, a high-speed measurement system was configured using a high-speed 8-bit projector, and a high-speed camera. The projector used was DynaFlash, the camera was acA640-750um (from Basler AG), and the PC was equipped with Intel ^®^Xeon ^®^CPU E5-2687W v4 and NVIDIA Quadro M5000 GPU. Using a frame rate of 500 fps, the projector with resolution set at 1024 × 768 and the camera with resolution set at 640 × 480 were synchronized. Figure 8 shows the system implemented and the experimental environment.

**Figure 9:**
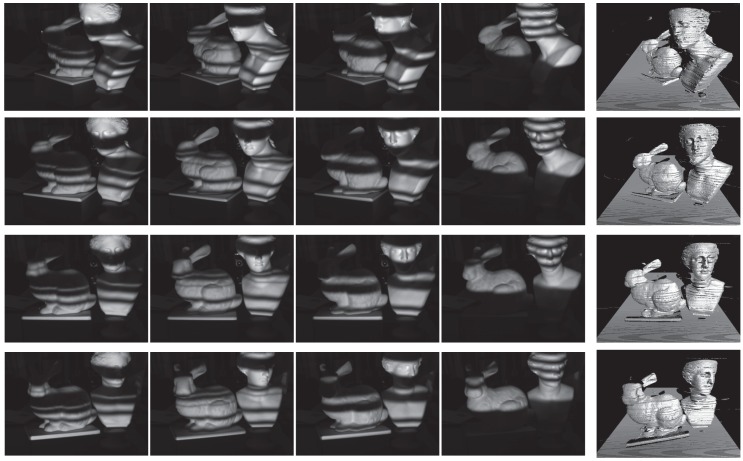
Measurement results in real environment. Each row shows the 3D shape reproduced using $I_{1}^{c}$, $I_{2}^{c}$, $I_{3}^{c}$, and $I_{4}^{c}$ (starting from left). From the data measured at 500 fps, data at 200 ms intervals were extracted. Starting from the top, the measurement results are shown at time *t* = 0, 200, 400, and 600 ms.

Here, the motion was kept at a level that can be manually generated, and because the environment had both noise and global illumination, the proposed four-step method was implemented. CPU processing was used only for control of projection by the projector and image capture by the camera, whereas GPU was used in the processing for calculating the phase from the images captured and calculating the 3D point group using triangulation and in the processing for displaying the obtained 3D point group implemented in shader using OpenGL. Because the processing time was 1.05 ms, which was well over the camera frame rate, real-time measurements at 500 fps could be performed. The measurement results are shown in Figure 9.

## 5. Discussion

First, as discussed in Section 4.1, it has been confirmed that in measuring moving objects, the fewer the number of projections, the lower would be the motion error. In particular, in *N* projection patterns $I_{n}^{\prime }\left(n=1\cdots N\right)$, there are very few terms that contain phase ϕ, and the smaller the time interval in terms containing phase ϕ the smaller the motion error. Moreover, the higher the velocity of the moving object, the more prominent would be the effect. Accordingly, when the velocity of the moving object is high, using the proposed three-step method, in which the number of projections and terms containing phase ϕ are fewer, it is possible to achieve highly accurate measurements.

Further, it was confirmed that for fewer number of projection periods, the effect of noise is strong, whereas the more the number of projection periods, the robustness against noise is also more. This is because, for larger number of periods, the effects on phase or projector coordinates corresponding to a deviation of 1 in intensity are smaller. Therefore, using larger number of periods in the patterns would lead to better measurement accuracy. However, the projector resolution has limitations; thus, having a certain number of periods can be sufficient. For the proposed three-step method, in which only periods up to 6π can be used, the effect of noise is strong because of insufficient number of periods; whereas, for the proposed four-step method, in which periods up to 24π can be used, robust measurements against noise could be performed, thereby suggesting that the number of periods was sufficient.

Moreover, as discussed in Section 4.2, even under conditions of intensity changes because of global illumination, robust measurements could be performed by the proposed method, which is not possible with the 2 + 2 method. This is because, in the proposed method, the average partial (at different regions) intensity for *N* projections is designed to be constant. Furthermore, while the intensity value is directly used in the 2 + 2 method, the proposed method is based on the relative magnitude of the intensity, in which stable phase unwrapping can be achieved, even with intensity changes because of global illumination. However, in the phase-shift method, the calculation of relative phase is affected by global illumination. Here, as expressed in Equations (7) and (12), difference and proportion are used to eliminate the effect of ambient light and reflectance ratio of the target measurement object. However, since the projection pattern changes for each frame, the light reflected at the target measurement object because of the projected pattern changes in each image captured. Accordingly, total elimination cannot be achieved, resulting in error. In the proposed method also, where Equations (22) and (32) are used, the generation of error in relative phase cannot be avoided.

Moreover, in the proposed method, pixelwise calculations are undertaken independently for calculating the absolute phase from the captured images. This enables stable measurement without being affected by the number of measurement targets or discontinuities. In addition, this is suitable for parallel processing using GPU, and as mentioned in Section 4.3, using the actual system, a processing time of 1.05 ms could be achieved. This way real-time measurement with a throughput of 500 fps was realized.

Furthermore, in Figure 6, Figure 7 and Figure 9, there are loss and noise like stripes, which did not occur in the conventional methods. That is caused by ambiguity of the tuple identification in the connection point. It may be easy to remove the noise and fill the loss by filtering.

As discussed above, for moving objects under global illumination conditions, using the proposed method, stable phase unwrapping can be achieved, and high-speed 3D measurements can be performed. It is to be noted that subtle errors in relative phase because of global illumination or motion error could not be completely suppressed. However, the ordered structure incorporated in the proposed method is not just limited to Equations (16)–(18) and Equations (24)–(27) used in the study, but can also be incorporated in other different patterns as well. For example, it could be effective that uses the coprime pair used in the conventional multi-frequency approaches. Accordingly, it is also possible to create a new design by selecting patterns based on robustness against effects of motion, necessary number of periods, and global illumination. Moreover, by combining with conventional motion error correction methods, it is likely that these errors can be suppressed further.

## 6. Conclusions

This paper proposed a phase-shift method for pixelwise phase unwrapping using a minimum of three steps by introducing a structured order, in which the project patterns are rearranged in each period. This method can be used with fewer steps; thus, it can be applied to moving objects. Further, compared with the conventional methods that are applicable to moving objects, the proposed method can achieve even more robust measurements against global illumination because low-frequency projection patterns are not used in it. Using the simulation and real environment experiments, it is confirmed that the proposed method can achieve highly accurate measurements of moving objects, even under environmental conditions in which noise and global illuminations are generated. Moreover, the processing time was about 1ms; thus, by implementing a high-speed, high-tone projector and a high-speed camera in a system, real-time measurements at 500 fps were achieved.

## Figures and Tables

**Table 1:** Continuity table for proposed method (three-step). hash(κ) denotes the movement destination in Figure 2d corresponding to index κ in Figure 2c.

κ in Figure 2c	1	2	3	4	5	6
hash (κ)	1	4	2	3	5	6

**Table 2:** Decoding in proposed method (three-step).

	$I_{3}^{c}<I_{1}^{c}<I_{2}^{c}$	$I_{2}^{c}<I_{1}^{c}<I_{3}^{c}$	$I_{3}^{c}<I_{2}^{c}<I_{1}^{c}$	$I_{1}^{c}<I_{2}^{c}<I_{3}^{c}$	$I_{2}^{c}<I_{3}^{c}<I_{1}^{c}$	$I_{1}^{c}<I_{3}^{c}<I_{2}^{c}$
tuple	(1,2,3)	(1,3,2)	(2,1,3)	(2,3,1)	(3,1,2)	(3,2,1)
κ in Figure 2c	1	2	3	4	5	6

**Table 3:** Table for continuity in proposed method (four-step). hash(κ) denotes the motion destination in Figure 3d corresponding to index κ in Figure 3c.

κ in Figure 3c	1	2	3	4	5	6	7	8	9	10	11	12	13	14	15	16
hash(κ) in Figure 3d	1	2	3	4	33	14	15	16	13	34	27	24	10	8	5	6
κ in Figure 3c	17	18	19	20	21	22	23	24	25	26	27	28	29	30	31	32
hash(κ) in Figure 3d	7	11	12	9	21	22	23	28	25	46	47	20	17	30	31	44
κ in Figure 3c	33	34	35	36	37	38	39	40	41	42	43	44	45	46	47	48
hash(κ) in Figure 3d	41	42	43	32	37	38	39	40	29	18	19	48	45	26	35	36

**Table 4:** Decoding in proposed method (four-step). Based on condition of $I_{\max}^{c}$ and $I_{\min}^{c}$, the corresponding tuple and $I_{\sin}^{c}$ and $I_{\cos}^{c}$ are determined. Moreover, the tuple index $\kappa_{\mathrm{tuple}}$ is also similarly determined.

$I_{\max}^{c}$	$I_{3}^{c}$	$I_{4}^{c}$	$I_{4}^{c}$	$I_{1}^{c}$	$I_{1}^{c}$	$I_{2}^{c}$	$I_{2}^{c}$	$I_{3}^{c}$	$I_{2}^{c}$	$I_{4}^{c}$	$I_{3}^{c}$	$I_{1}^{c}$
$I_{\min}^{c}$	$I_{4}^{c}$	$I_{3}^{c}$	$I_{1}^{c}$	$I_{4}^{c}$	$I_{2}^{c}$	$I_{1}^{c}$	$I_{3}^{c}$	$I_{2}^{c}$	$I_{4}^{c}$	$I_{2}^{c}$	$I_{1}^{c}$	$I_{3}^{c}$
tuple	(1,2,3,4)	(1,2,4,3)	(4,1,2,3)	(3,1,2,4)	(3,4,1,2)	(4,3,1,2)	(1,3,4,2)	(1,4,3,2)	(1,3,2,4)	(1,4,2,3)	(4,1,3,2)	(3,1,4,2)
$I_{\sin}^{c}$	$I_{1}^{c}$	$I_{1}^{c}$	$I_{2}^{c}$	$I_{2}^{c}$	$I_{3}^{c}$	$I_{3}^{c}$	$I_{1}^{c}$	$I_{1}^{c}$	$I_{1}^{c}$	$I_{1}^{c}$	$I_{2}^{c}$	$I_{2}^{c}$
$I_{\cos}^{c}$	$I_{2}^{c}$	$I_{2}^{c}$	$I_{3}^{c}$	$I_{3}^{c}$	$I_{4}^{c}$	$I_{4}^{c}$	$I_{4}^{c}$	$I_{4}^{c}$	$I_{3}^{c}$	$I_{3}^{c}$	$I_{4}^{c}$	$I_{4}^{c}$
$\kappa_{\mathrm{tuple}}$	0	1	2	3	4	5	6	7	8	9	10	11
